# Comparative phenotypic profiling of the JAK2 inhibitors ruxolitinib, fedratinib, momelotinib, and pacritinib reveals distinct mechanistic signatures

**DOI:** 10.1371/journal.pone.0222944

**Published:** 2019-09-27

**Authors:** Jack W. Singer, Suliman Al-Fayoumi, Jason Taylor, Sharlene Velichko, Alison O’Mahony

**Affiliations:** 1 CTI BioPharma Corp., Seattle, Washington, United States of America; 2 Elson Floyd College of Medicine, Washington State University, Seattle, Washington, United States of America; 3 Eurofins Discovery, Phenotypic Services, Burlingame, California, United States of America; National Cancer Institute, UNITED STATES

## Abstract

Janus kinase-signal transducers and activators of transcription (JAK-STAT) signaling is critical to multiple cellular processes, including survival, differentiation, and proliferation. JAK-STAT signaling dysregulation has been noted in inflammatory disorders, and aberrant JAK2 pathway activation has been implicated in myelofibrosis and polycythemia vera. Moreover, 4 therapeutic JAK2 inhibitors (ruxolitinib, fedratinib, momelotinib, and pacritinib) have either been approved or are in advanced clinical development for myelofibrosis. Although all inhibit JAK2, reports indicate that they also inhibit other kinases. Profiling based solely on *in vitro* potencies is insufficient to predict the observed clinical effects. To provide further translational insights into clinical outcomes, we compared phenotypic biomarker profiles of ruxolitinib, fedratinib, momelotinib, and pacritinib in the BioMAP^®^ Diversity PLUS panel of 12 human primary cell systems designed to recapitulate key aspects of tissue and disease states. Biomarker activity profiles that represent mechanistic signatures for each agent were compared with each other and a database of reference benchmark profiles. At clinically relevant concentrations, these agents had distinct biomarker impacts indicating diverse mechanistic signatures, suggesting divergent clinical effects for each agent. They disparately modulated inflammatory cytokine production and immune function. At clinically relevant concentrations, ruxolitinib had the broadest scope of activities across all 12 cellular systems, whereas pacritinib was more specific for the BT system (modelling T cell-dependent B cell activation) and exhibited the strongest inhibition of sIL-17A, sIL-2, and sIL-6. All 4 agents were antiproliferative to B cells, but ruxolitinib and momelotinib were also antiproliferative to T cells. These differential activities likely reflect distinct secondary pharmacology for these agents known primarily as JAK2 inhibitors. The phenotypic analysis reported herein represents key data on distinct modes-of-action that may provide insights on clinical outcomes reported for these agents. Such translational findings may also inform the development of next-generation molecules with improved efficacy and safety.

## Introduction

The Janus kinase-signal transducers and activators of transcription (JAK-STAT) signaling pathways mediate cellular responses and influence cell survival, differentiation, and proliferation [1–3]. Dysregulated JAK-STAT signaling has been implicated in a variety of inflammatory diseases [4–6]. In 2005, the discovery of the constitutively activating *JAK2V617F* mutation in the majority (97%) of patients with polycythemia vera (PV) and approximately 50% of patients with idiopathic myelofibrosis (MF) confirmed the central role played by JAK2 in the pathogenesis of myeloproliferative neoplasms [7–9]. As a consequence of identification of a disease-specific activating mutation, several JAK2 inhibitors were identified and entered development. The first to be approved was ruxolitinib, a JAK1/2 inhibitor that was approved by the FDA in 2011 for patients with intermediate or high-risk MF. Although not specifically contraindicated, ruxolitinib is not recommended for patients with a baseline platelet count <50 × 10^9^L [10, 11]. Its approval was based on results of the COMFORT-I (ruxolitinib versus placebo) and COMFORT-II (ruxolitinib versus best available therapy [BAT]) trials in patients with intermediate-2 or high-risk primary MF, post-PV MF, or postessential thrombocythemia MF (post-ET MF) [12–14]. Subsequently, other JAK2 inhibitors were identified, and the 3 that were co-evaluated in this study include fedratinib [15], momelotinib [16], and pacritinib [17], all currently in advanced clinical development.

Although JAK2 is the primary pharmacological target of ruxolitinib, momelotinib, pacritinib, and fedratinib, each agent differs with respect to inhibition of other kinases [18–21]. These secondary-target effects arise as a consequence of the highly conserved nature of kinase ATP-binding pockets [22]. Among these agents, only pacritinib does not inhibit JAK1 at physiologically relevant concentrations and therefore does not directly suppress signaling by interferons and IL-6 [20]. Pacritinib appears to exert its anti-inflammatory effects upstream of JAK1 through inhibition of IRAK1 and suppression of downstream inflammatory cytokine production [23–25].

Differences in kinase inhibitor profiles may ultimately underlie differences in off- target effects, efficacy, or specific indications, as has been the case for imatinib [26]. However, translating *in vitro* preclinical pharmacology into expected pharmacological effects in humans remains a challenge. Translational studies using intact, complex human cellular systems may provide improved insights into the differential clinical effects of drugs. The BioMAP^®^ phenotypic profiling platform (Eurofins Pharma Discovery Services [EPDS], Burlingame, CA) combines human phenotypic assays and specialized data analytics to evaluate the impact of a test agent in complex models of human tissue and disease biology [22–25]. In this study, the Diversity PLUS^™^ panel was used to test 4 JAK2 inhibitors, ruxolitinib, momelotinib, pacritinib, and fedratinib, at clinically relevant concentrations. This panel consists of 12 individual systems constructed with one or more tissue-specific human primary cell types from pooled healthy donors that are stimulated and used to measure impacts on assay endpoints selected for biological and therapeutic relevance [27–30]. For each inhibitor, the cumulative changes in biomarker readouts (above or below baseline) were used to generate a BioMAP profile for each tested concentration, which was then compared with the other agents tested as well as the profiles of more than 4000 benchmarks in the BioMAP Reference Database.

## Materials and methods

### Materials

Pacritinib was provided by CTI BioPharma Corp. (Seattle, WA, USA). Ruxolitinib, fedratinib, and momelotinib were obtained from Selleckchem (Houston, TX, USA). Primary human endothelial cells (HuVEC), neonatal foreskin fibroblasts (HDFn), bronchial epithelial cells (BE), coronary arterial smooth muscle cells (CASMC), and keratinocytes (HEK) were obtained from Lonza, Lifeline Cell Technologies and Cell Applications. Peripheral blood mononuclear cells (PBMCs) and positively selected primary normal human CD19+ B cells, CD4+ T cells, and CD14+ monocytes were purchased (AllCells, Emeryville, CA) or were isolated in-house (LeukoPak [StemCell and Physicians Plasma Alliance]). All primary cells were obtained under protocols that were reviewed by Institutional Review Board(s) that operate in accordance with the requirement of EPA Regulation 40 CFR 26 and HHS Regulation 45 CFR 46 of the US Department of Health and Human Resources for the protection of human research subjects. Stimuli include recombinant human IFNγ, TNFα, and IL-1β, IL-4, PDGF, FGF, EGF, and TGFβ (Peprotech, SEB, and TSST [Toxin Technologies]) Histamine, LPS, (Sigma), anti-human IgM (Southern Biotechnology), and Zymosan (Invivogen). Mouse detection antibodies against human biomarkers were obtained from commercial sources, including BD BioSciences, Sigma, and R&D Systems.

### Methods

#### Cell culture

**Fig 1:**
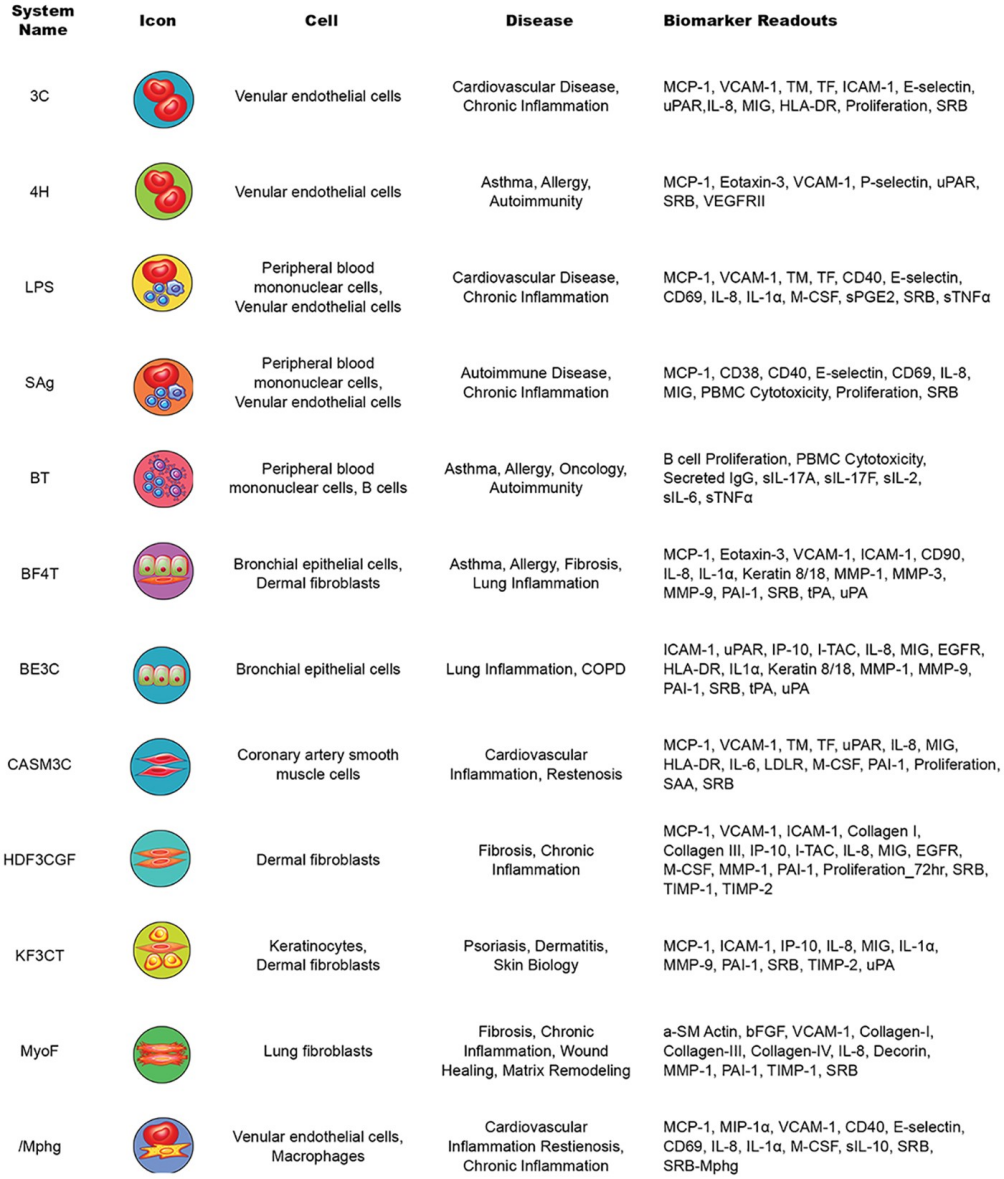
BioMAP Diversity PLUS panel system descriptions. bFGF, basic fibroblast growth factor; CD, cluster of differentiation; COPD, chronic obstructive pulmonary disease; EGFR, epidermal growth factor receptor; HLA-DR, human leukocyte antigen–antigen D related; ICAM-1, intercellular adhesion molecule-1; IgG, immunoglobulin G; IL, interleukin; IP-10, interferon gamma-induced protein 10; I-TAC, interferon-inducible T-cell alpha chemoattractant; MCP-1, monocyte chemoattractant protein-1; M-CSF, macrophage colony-stimulating factor; MIG, monokine induced by interferon gamma; MMP, matrix metalloproteinase; PAI-1, plasminogen activator inhbitor-1; PBMC, peripheral blood mononuclear cell; sPGE2, soluble prostaglandin E2; sTNFα, soluble tumor necrosis factor α; TIMP, tissue inhibitor of metalloproteinases; tPA, tissue plasminogen activator; uPA, urokinase-type plasminogen activator; uPAR, urokinase receptor; VCAM-1, vascualr cell adhesion molecule-1; VEGFRII, vascular endothelial growth factor receptor II.

Detailed protocols for the BioMAP primary human cell culture and coculture systems have previously been published [31–34]. The 12 systems in the Diversity PLUS panel (Fig 1) were used to test agents across a broad set of systems modeling different human disease states. Systems constructed with one or more primary cell types from normal human donors stimulated with cytokines or growth factors recapitulate relevant signaling networks that naturally occur in human tissue or disease states. Systems model vascular biology in Th1-type (3C; HUVEC) and Th2-type (4H; HUVEC) inflammatory environments as well as in a Th1-type inflammatory state specific to arterial smooth muscle cells (CASM3C; coronary artery smooth muscle cells); systemic immune response including monocyte-driven Th1 inflammation (LPS; PBMC and HUVEC) or T-cell stimulation (SAg; PBMC and HUVEC), chronic Th1 inflammation driven by macrophage activation (*I*Mphg; HUVEC and macrophages), and the T cell-dependent activation of B cells occurring in germinal centers (BT; CD19^+^ B cells and PBMC); Th1 (BE3C; bronchial epithelial cells), and Th2 (BF4T; bronchial epithelial cells and HDFn) airway inflammation of the lung; myofibroblast-lung tissue remodeling (MyoF; differentiated lung myofibroblasts); and skin biology including Th1 cutaneous inflammation (KF3CT; keratinocytes and HDFn) and wound healing (HDF3CGF; HDFn). Biomarkers were selected for therapeutic and biologic relevance and were validated using agents with known mechanisms of action. Systems were stimulated in the presence of the test agent and incubated for 24 hours except for the BT system (incubated for 72 hours for all readouts except IgG, which is measured at 6 days) and MyoF (incubated for 48 hours).

#### Endpoint measurements

Cells were plated in 96-well plates, containing the test compounds, appropriate drug controls, negative controls, and vehicle controls. Compounds were dissolved in DMSO (0.1% final concentration) and diluted to final concentration as indicated. Phenotypic activity profiles for the 4 concentrations of each compound were generated, and key biomarker changes were assessed. Test compounds were added 1 hour before stimulation of the cells and were present during the entire stimulation period. Biomarker levels of cell-associated and cell membrane targets were assessed by direct ELISA. Soluble end points were quantified from supernatants by using homogeneous time-resolved fluorescence (HTRF^®^) detection, bead-based multiplex immunoassays, or capture ELISA. Overt adverse effects on cell proliferation and viability were measured using sulforhodamine B (Millipore-Sigma, MO, USA) for adherent cells and alamarBlue^®^ (Bio-Rad, CA, USA) for suspension cells. For proliferation assays, the individual cell types were cultured at subconfluence and measured relative to the vehicle control samples tested in parallel at time points optimized for each system (48–96 hours).

#### Statistical analysis

Biomarker measurements for each test agent-treated samples were divided by the average of vehicle control samples (at least 6 vehicle controls from the same plate) to generate a log_10_-transformed ratio. Significance envelopes using historical vehicle control data were used as a 95% confidence interval. Biomarker activities were annotated when 2 or more consecutive concentrations of the test agent were outside of the significance envelope with an effect size >20% compared with the vehicle control (|log_10_ *ratio*| >0.1) for each system. Cytotoxicity was flagged when total protein levels (SRB readout) decreased by more than 50% (log_10_ *ratio* of SRB <-0.3) and were indicated by a thin black arrow above the X-axis. Concentrations of test agents with detectable broad cytotoxicity were excluded from biomarker activity annotation and downstream benchmark analysis. Cytotoxic arrows only required one concentration to meet the indicated log_10_ ratio threshold for profile annotation. The profile of one concentration of a test agent was compared using overlay analysis to that of another compound from the same experiment or from the BioMAP reference database. Common biomarker readouts were identified when the readouts for both profiles were outside of the significance envelope in the same direction with an effect size >20% (|log_10_ *ratio*| >0.1). Differentiating biomarkers were annotated when 1 profile had an activity outside the significance envelope with an effect size >20% (|log_10_ *ratio*| >0.1), and the readout for the other profile was either inside the envelope or in the opposite direction. BioMAP assay acceptance criteria included the multiparameter data sets generated by the BioMAP platform for agents tested in the specific BioMAP systems. Assays contained drug controls (eg, legacy control test agent colchicine), negative controls (eg, nonstimulated conditions), and vehicle controls (eg, DMSO) appropriate for each system. BioMAP assays were plate-based, and data acceptance criteria depended on both plate performance (% CV of vehicle control wells) and system performance across historical controls for that system. The QA/QC Pearson Test was performed by first establishing the 1% false-negative Pearson cutoff from the reference dataset of historical positive controls. The process iterated through every profile of system biomarker readouts in the positive control reference dataset, calculating Pearson values between each profile and the mean of the remaining profiles in the dataset. The overall number of Pearson values used to determine the 1% false-negative cutoff was the total number of profiles present in the reference dataset. The Pearson value at the one percentile of all values calculated was the 1% false-negative Pearson cutoff. A system passed if the Pearson value between the experimental plate’s negative control or drug control profile and the mean of the historical control profiles in the reference dataset exceeded this 1% false-negative Pearson cutoff. Overall assays were accepted when each individual system passed the Pearson test and 95% of all project plates had % CV <20%.

Extent of similarity between compound profiles was determined using a custom similarity metric. A Pearson’s correlation coefficient (*r*) was generated to measure the linear association between profiles based on similarity in direction and magnitude of the relationship. A per-system weighted average Tanimoto metric was used as a filter to account for underrepresentation of less robust systems. Based on the optimal performance of reference compounds, profiles were identified as having mechanistically relevant similarity if *r* ≥ 0.7. A Fisher *r*-to-*z* transformation was used to calculate a z-score to convert a short tail distribution into a normal distribution. Finally, a BioMAP Z-standard, which adjusts for the number of common readouts (CRs) is generated according to the formula Z-Standard = *z* √(*CR −* 3). A larger value corresponds to a higher confidence level, and this metric is used to rank similarity results.

Functional clustering of agent profiles used Pearson correlation values for pairwise comparisons of profiles for each agent at each concentration, and then subjected the pairwise correlation data to multidimensional scaling. Similar profiles with *r* ≥ 0.7 were connected by lines. Agents not clustering with one another were interpreted as mechanistically distinct. Mechanistic HeatMAP profiles were calculated by averaging the value for each biomarker endpoint for all profiles selected (multiple agents at different concentrations) to build the consensus mechanism profile. Biomarker activities were colored in the heatmap for consensus mechanism and compounds when they had expression relative to vehicle controls outside the significance envelope.

## Results

**Fig 2:**
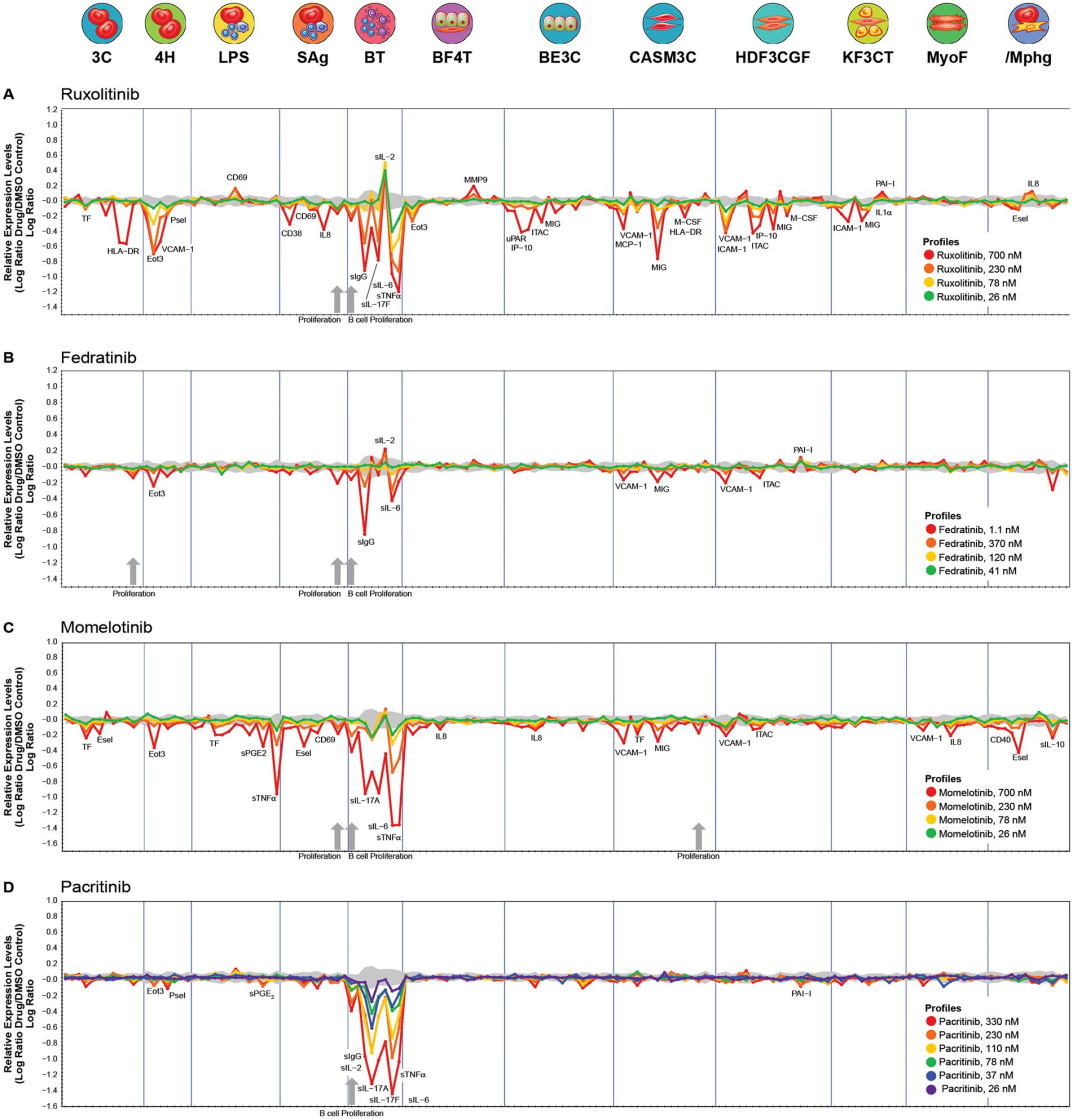
BioMAP biomarker profiles for (A) ruxolotinib, (B) fedratinib, (C) momelotinib, and (D) pacritinib. X-axes list the quantitative protein biomarker readouts as well as proliferation and viability endpoints measured in each system. Y-axes show log-transformed ratios of biomarker readouts for each test agent/vehicle controls. The grey region around the Y-axis shows the 95% significance envelope generated from historical vehicle controls used as a confidence interval to identify activities of each test agent. Biomarker activities are annotated when ≥2 consecutive concentrations change in the same direction relative to vehicle controls, are outside of the significance envelope, and have ≥1 concentration with an effect size >20%. Biomarker key activities are described as modulated if these activities increase in some systems, but decrease in others. Cytotoxicity is indicated on the profile plot by a thin black arrow above the X-axis, and antiproliferative effects are indicated by a thick grey arrow. X axis labels are defined in Table 1.

BioMAP Diversity PLUS testing utilizes 148 biomarker readouts (7–17 per system) selected for therapeutic and biological relevance which are predictive for disease outcomes or specific drug effects and are validated using agents with known mechanisms of action [29, 30, 33]. Biomarker activities for ruxolitinib, fedratinib, momelotinib, and pacritinib in these systems at the indicated test concentrations are shown in Fig 2A–2D. Distinct biomarker profiles reflecting different mechanistic signatures for each agent were noted. Compounds were first assessed for impact on the proliferative response of multiple primary cell types represented in this panel (Table 1). The highest concentration of pacritinib (700 nM) decreased the PBMC-cytotoxicity endpoint below the determined threshold of ≥50% reduction in PBMC viability relative to vehicle control levels (|log_10_ *ratio* | <-0.3) in the SAg and BT systems, and therefore only data for the lower nontoxic concentrations were included in this analysis. Momelotinib at 700 nM also decreased the viability of PBMC but did not reach the determined threshold. Fedratinib (1.1 uM) did not affect PBMC viability.

**Table 1:** Summary of antiproliferative and biomarker activities for ruxolotinib, federatinib, momelotinib, and pacritinib in the BioMAP Diversity Plus panel. All concentrations are expressed in nM units.

Test Agents	Concentrations	Detectable Cytotoxicity^a^	Antiproliferative Effects^b^	Inflammation-Related Activities^c^	Immunomodulatory Activities^c^	Tissue Remodeling Activities^c^	Hemostasis-Related Activities^c^
Ruxolitinib	700, 230, 78,26	None	B cells (700, 230), T cells (700, 230, 26)	↓ Eotaxin 3, E-selectin, VCAM-1, MCP-1, sTNFα, I-TAC, ICAM-1, MIG, IP-10, IL-1α, P-selectin ⇵ IL-8	↓sIgG, M-CSF, HLA-DR, CD38, sIL-6, sIL-17F ↑sIL-2 ⇵CD69	↓uPAR ↑ PAI-1, MMP-9	↓TF
Fedratinib	1100, 370, 120, 41	None	B cells (1100) T cells (1100) Endothelial cells (1100)	↓Eotaxin 3, VCAM-1, I-TAC, MIG	↓sIgG, sIL-6 ↑ sIL-2	↑PAI-1	None
Momelotinib	700, 230, 78, 26	None	B cells (700, 230) T cells (700) CASM cells (700)	↓Eotaxin 3, E-selectin, VCAM-1, sTNFα, I-TAC, MIG, IL-8, sPGE_2_	↓CD40, sIL-10, sIL-17A, sIL-6, CD69	None	↓TF
Pacritinib	700, 330, 230, 110, 78, 37, 26	PBMC (SAg and BT; 700)	B cells (330, 230 110, 78)	↓ sTNFα	↓sIgG, sIL-17A, sIL-6, sIL-17F, sIL-2 ↑CD69	↓ MMP9	None

^a^ (system; concentration)

^b^ (concentration)

^c^ ↑ Indicates an increase relative to vehicle control; ↓indicates a decrease relative to vehicle control; ⇵ indicates an increase in some but not in others relative to vehicle control.

At noncytotoxic concentrations, pacritinib selectively blocked the proliferation of human primary B cells in the BT system over a range of test concentrations (330 nM, 230 nM, 110 nM, and 78 nM). In contrast, ruxolitinib, momelotinib, and fedratinib were more broadly antiproliferative on multiple human primary cell types at the indicated concentrations. Specifically, momelotinib blocked the proliferation of human primary B cells (700 nM, 230 nM), T cells (700 nM), and coronary artery smooth muscle cells (700 nM). Ruxolitinib was antiproliferative to human primary B cells (700 nM, 230 nM) and T cells (700 nM, 230 nM, 26 nM), and fedratinib was antiproliferative to B cells (1.1 uM), T cells (1.1 uM), and endothelial cells (1.1 uM).

In addition to the antiproliferative effects, these 4 drugs differed in the overall scope of impacts measured across 148 biomarkers classified based on biological and disease relevance (Table 1). Over the indicated concentrations, pacritinib (330 nM, 220 nM, 110 nM, 78 nM, 37nM, and 26 nM) demonstrated a total of 10 annotated activities, whereas fedratinib (1100 nM, 370 nM, 120 nM, and 41 nM) had 9 annotated activities. In contrast, both momelotinib (700 nM, 230 nM, 78 nM, and 26 nM) and ruxolitinib (700 nM, 230 nM, 78 nM, and 26 nM) were more broadly active with 23 and 38 annotated biomarker activities, respectively.

Pacritinib activities were detected in only 4 systems with the majority of impacts observed in the BT system modeling T cell-dependent B cell activation that occurs in germinal centers of secondary lymphoid organs. Indeed, all concentrations of pacritinib were active in this system where biomarkers of both early B cell activation, (sIL-17A, sIL-6, sIL-17F, and sIL-2) and plasma cell differentiation (sIgG) were strongly inhibited in a dose-dependent manner. Higher concentrations of pacritinib (330 nM and 230 nM) were less selective, with modest effects detected outside the BT system, including decreased Eotaxin 3 and P-selectin in the 4H system modeling Th2-type vascular inflammation and sPGE_2_ in the LPS system modeling monocyte activation. These high exposures also weakly inhibited the tissue remodeling biomarker PAI-1 in the HDF3CGF system modeling wound healing biology.

At comparable exposures, fedratinib (370 nM, 120 nM, 41 nM, and 14 nM) was inactive in the Diversity PLUS panel, with no activities that met the defined criteria for annotation. Inclusion of a higher concentration of fedratinib (1.1 μM) revealed 9 activities that were annotated at the top 2 concentrations only in 4 systems, including decreased sIgG and sIL-6 and increased sIL-2 in the BT system without any impacts on sIL-17A, sIL-17A, or TNFα. At these exposures, fedratinib also modestly decreased the inflammation-related eotaxin 3, VCAM-1, I-TAC, and MIG biomarkers as well as increased the tissue remodeling biomarker PAI-1. Together these data indicate that at equimolar exposures, fedratinib compared with pacritinib is a less selective inhibitor of B cell biology.

Although a total of 23 momelotinib activities met the annotation criteria, a sharp dose response was observed between the top 2 concentrations, suggesting secondary targets of the highest concentration of 700 nM. When this concentration was excluded from the analysis, momelotinib had only 6 annotated activities with the 230 nM, 78 nM, and 26 nM doses. These effects included decreased levels of the inflammation biomarker TNFα in both the LPS and BT systems, inhibition of sIL-17A and sIL-6 (BT system), and modestly decreased TF in the 3C and CASM3C systems. Unlike pacritinib, momelotinib did not impact the other immune biomarkers in the BT system at comparable concentrations.

Ruxolitinib was the most broadly active with 38 annotated readouts detected in 11 of the 12 systems in this profiling panel. Ruxolitinib demonstrated immunomodulatory impacts in the BT system (decreased sIL-6 and sIL-17F and increased sIL-2) (Table 1). Ruxolitinib also decreased the inflammation biomarkers M-CSF and HLA-DR in the CASM3C and HDF3CGF systems and the hemostasis-related biomarker TF in the 3C system. Ruxolitinib impacted multiple inflammation-related activities, including decreased eotaxin 3, VCAM-1, MCP-1, sTNFα, I-TAC, MIG, IP-10, IL-1α, P-selectin, and increased IL-8 levels. Taken together, these data indicate that of these 4 agents, pacritinib is the most potent in blocking B-cell proliferation, activation, and differentiation. It also displayed the greatest selectivity against other cellular systems and is therefore less likely to manifest adverse outcomes related to impacts on other tissue cell types.

**Fig 3:**
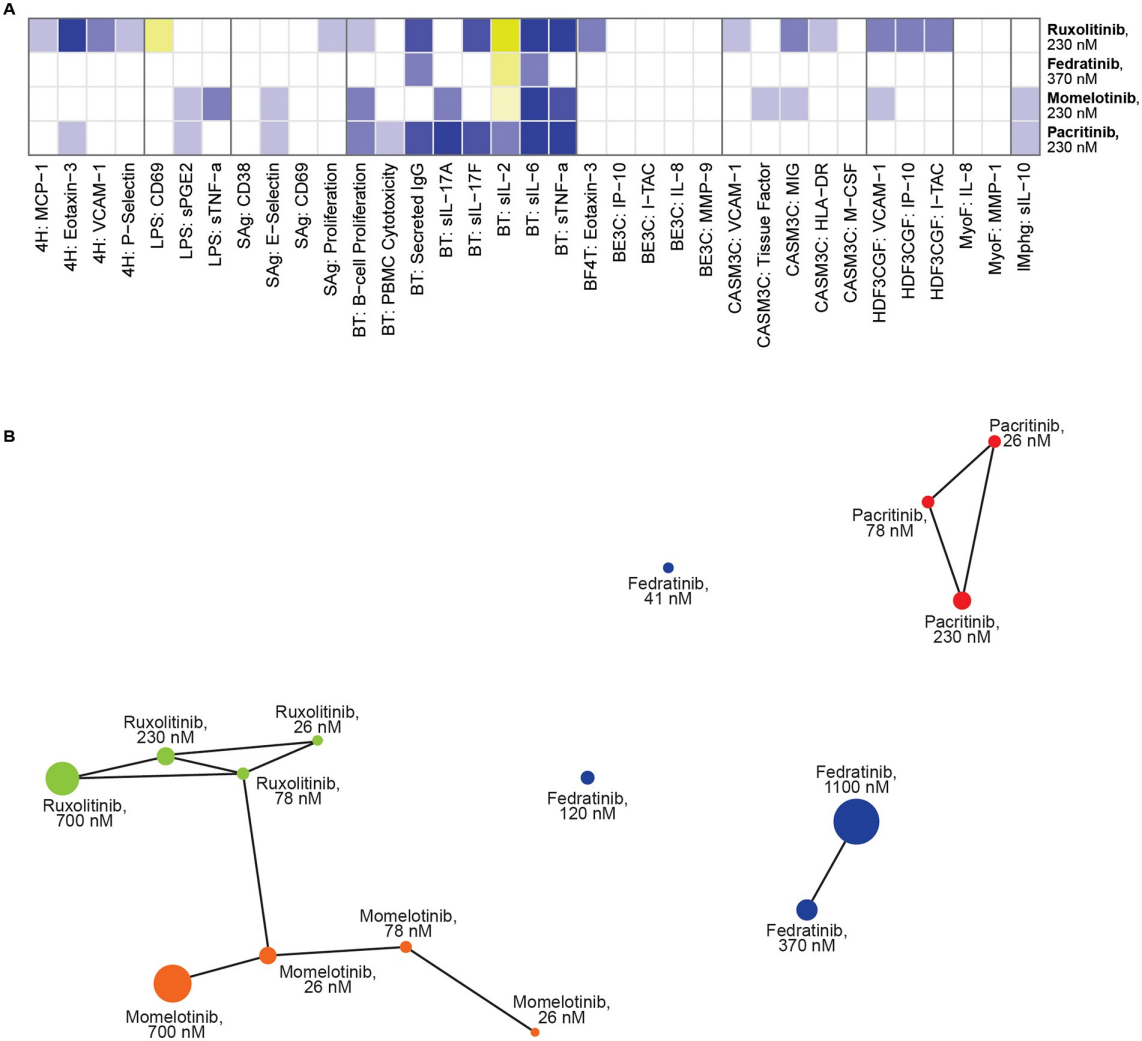
(A) Heat map of fedratinib, momelotinib, pacritinib, and ruxolitinib activities in the BioMap Diversity Plus panel. Blue indicates decreased expression and yellow indicates increased expression. Darker shades represent greater change in biomarker activity relative to vehicle control. (B) Cluster map from pairwise correlation analysis of ruxolotinib, fedratinib, momelotinib, and pacritinib.

Comparative HeatMAP analysis (Fig 3A) of all agents at their second tested concentrations of 230 nM or 370 nM revealed that only sIL6 in BT was commonly decreased by all 4 agents. Pacritinib shared the following activities with the other tested agents at these similar exposures: momelotinib and pacritinib decreased sPGE2 (LPS), E-selectin (SAg), B-cell proliferation, sIL-17A, sIL-6, sTNFα (BT), and sIL-10 (*l*Mphg). These 2 agents could be differentiated based on the following activities: momelotinib but not pacritinib decreased TF (3C and CASM3C), sTNFα (LPS), MIG (CASMC), and VCAM-1 (HDF3CGF). Pacritinib and ruxolitinib at the 230 nM concentration shared 6 common activities that decreased within the indicated systems: eotaxin 3 (4H) and B-cell proliferation, sIgG, sIL-17F, sIL-6, sTNFα (BT). However, these agents had a total of 19 differentiating activities annotated, indicating that these 2 agents act by distinct mechanistic pathways. Pacritinib (330 nM) and fedratinib (370 nM) shared only 2 common activities: decreased sIgG and sIL-6 in the BT system; however, a total of 13 differentiating activities were detected. Notably, pacritinib treatment resulted in a major reduction in sIL-2 levels in the BT system, whereas ruxolitinib, fedratinib, and momelotinib increased sIL2 levels, suggesting that this biomarker may be a sentinel compensatory response biomarker for JAK1/3 inhibition. Using pairwise correlation analysis (Fig 3B) to cluster the most similar profiles at a Pearson’s correlation coefficient *r* ≥0.8 revealed that momelotinib and ruxolitinib clusters connected, indicating they have overlapping mechanisms. In contrast, pacritinib and fedratinib clustered only across their concentrations, indicating that the phenotypic signature of each agent is distinct but is maintained across the range of treatment exposures.

In an unsupervised search for mathematically similar compound profiles from the BioMAP Reference Database, the profile of pacritinib at a clinically relevant concentration (230 nM) was found to be highly similar to multiple concentrations of the PI3Kδ inhibitor idelalisib as well as other PI3K inhibitors, pictilisib (GDC0941;120 nM) and IC87114 (120 nM). All profile matches had Pearson’s correlation coefficients where *r* >0.9, which is above the determined threshold for mechanistic similarity (*r* >0.7). The top 4 similarity matches for pacritinib were idelalisib at 110 nM, 330 nM, 3000 nM, and 1000 nM. These profiles were also top matches for the other tested concentrations of pacritinib. Together, these data indicate that pacritinib and selective PI3K inhibitors have overlapping phenotypic signatures that may lead to common biological impacts. Ruxolitinib (230 nM) was most similar to profiles for other JAK inhibitors, including baracitinib (120 nM and 370 nM), filgotinib (3.3 uM), and tofacitinib (1.1 uM) (*r* >0.7) again indicating mechanistic similarity. Top matches for fedratinib (370 nM) included the JAK inhibitors tofacitinib at 41 nM and ruxolitinib at 14 nM with Pearson’s correlation coefficients of *r* >0.7, confirming their shared mechanism. In contrast, momelotinib (230 nM) had no similarity matches, indicating it either has a novel target/MoA or demonstrates polypharmacology that leads to a unique activity profile.

## Discussion

Understanding the basis for observed clinical differences among kinase inhibitors with identical primary targets remains challenging. Studies with cell lines or single-cell assays lack the complexity or translational relevance found in tissues and *in vivo* systems. In contrast, the BioMAP systems used herein consist of diverse human primary cell types pooled from multiple donors and used at low passage to preserve physiological regulatory networks that are stimulated under complex multifactorial conditions to recapitulate key aspects of tissue and disease states. These *in vitro* models are designed to preserve compensatory crosstalk and feedback mechanisms that are relevant to *in vivo* outcomes and thus can provide insights into pathway interventions and off-target activities. In addition, they enable head-to-head comparison of test agents for impacts on translational biomarkers used in clinical trials and are hypothesis generating for differential clinical effects of agents addressing overlapping targets [30, 33, 35, 36].

This study compared the BioMAP phenotypic profiles of 4 agents known primarily for their JAK2 inhibition that have been clinically investigated and show important clinical activity in the treatment of myelofibrosis. The *in vitro* potency of each of these agents against wild type and the common mutant JAK2 (V617F) is in the low nM range. Each of these agents has activities against other kinases, but nevertheless these target profiles provide only limited insights into their differential clinical effects. The present study of their effects in complex cellular systems was undertaken to provide translational insights into their *in vivo* effects.

Each agent was found to have a distinct phenotypic profile that was consistent across all noncytotoxic concentrations tested. For all agents, the greatest effects were observed in the BT system, but the extent to which biomarkers within that system were modulated varied considerably. Pacritinib had the most potent effect on reducing levels of the key proinflammatory cytokines, whereas fedratinib was the weakest agent. Serum levels of sIL-2, together with its receptor IL-2Rα, were significantly higher in patients with myeloproliferative diseases than in healthy controls [37] and were increased by ruxolitinib but decreased by pacritinib.

Further differentiation in the effects of these agents was seen in other systems. Ruxolitinib had the broadest scope of activities, with 38 annotated biomarkers detected, including those in systems modeling cardiovascular disease (3C, CASM3C) and airway inflammation (BE3C) (Fig 2A). Pacritinib had a more selective pattern of modulated biomarker levels with activities detected only in the BT system at all tested concentrations as well as limited modest effects in other systems detected only at the highest 2 concentrations. The antiproliferative profiles of the 4 kinase inhibitors proved unique as well. All 4 inhibitors were antiproliferative to B cells, but ruxolitinib and momelotinib also inhibited T-cell proliferation [38, 39]. The suppression of T-cell proliferation by ruxolitinib, likely through inhibition of JAK1 signaling by IL-2, may be associated with its increased rate of opportunistic infections *in vivo* [40]. Although pacritinib proved cytotoxic in the BT and SAg systems at a very high concentration (700 nM), clinical data indicate that peak systemic levels of free pacritinib are approximately 200 nM, and thus direct cytotoxic effects are unlikely to occur [41, 42].

In an unsupervised search of the BioMAP reference benchmark database containing >4800 compound profiles for the most similar profiles for all 4 test agents at relevant physiological concentrations, pacritinib was most similar to profiles of the PI3Kδ inhibitor idelalisib at 4 different concentrations. The top match for pacritinib (230 nM) was ideliasib (110 nM) with a Pearson’s correlation coefficient of *r* = 0.948, indicating highly similar mechanistic signatures and overlapping impacts on biology. Idelalisib has been approved [43] in relapsed chronic lymphocytic leukemia (in combination with rituximab in patients with limiting comorbidities), relapsed follicular lymphoma, and small lymphocytic lymphoma in the third and later lines of therapy. Other members of this target class of compounds, pictilisib and IC87114, were also found to have highly similar profiles, including selective antiproliferative effects on B cells and inhibition of IL-2 and IL-17 in the BT system, as previously reported [35]. Pacritinib has been shown to suppress 2 downstream targets of PI3K inhibitors, pERK and P38MAPK, through suppression of IRAK1 in AML cell lines and patient specimens [44]. In addition, *in vitro* studies have demonstrated high levels of cytotoxic activity for pacritinib in CLL cells [45].

Both fedratinib and ruxolitinib were mechanistically similar to compounds in the JAK inhibitor class, but fedratinib had a much more restricted similarity, matching only one low concentration of tofacitinib at 41 nM and ruxolitinib at 14 nM. In contrast, the ruxolitinib profile at 230 nM was similar to multiple concentrations of different JAK inhibitors, including baracitinib (120 nM and 370 nM), filgotinib (3.3 μM), and tofacitinib (1.1 μM) all with Pearson’s correlation coefficients above our threshold for mechanistic similarity.

The distinct phenotypic profiles for pacritinib, ruxolitinib, momelotinib, and fedratinib likely reflect the subtleties of biological effects of competitive ATP binding site agents developed against a single target, JAK2, that nevertheless affect other kinases in multiple target classes. Differential effects on nonprimary target kinases may be responsible for the differences in effects on biomarkers within the system of greatest interest, BT, which models T-cell dependent B cell activation, proliferation, and differentiation to antibody-producing plasma cells relevant to normal adaptive immunity as well as to the pathophysiology of B-cell lymphomas. These differential effects and activities in other systems also suggest that clinical outcomes may well be distinct for each agent. Although these phenotypic results do not directly identify precise pathway intervention points that give rise to observed biomarker activities, they do provide an informed starting point for further exploration and extrapolation of these data to the pathological clinical conditions that may suggest other potential disease targets for each of these agents.

## Supporting information

S1 File Underlying data used for analysis. (XLSX)
